# Supplementary material for: Pyruvate Dehydrogenase Kinase 1 inhibition mediated oxidative phosphorylation enhancement in cartilage promotes osteoarthritis progression
Source: BMC Musculoskelet Disord. 2023 Jul 20;24:597. doi: 10.1186/s12891-023-06585-6 (PMC10357736; doi:10.1186/s12891-023-06585-6)
Supplement: Supplementary file 1 — Additional file 1. [file 12891_2023_6585_MOESM1_ESM.zip › BP.Gsea.1653623667859/GOBP_REGULATION_OF_DNA_TEMPLATED_TRANSCRIPTION_IN_RESPONSE_TO_STRESS.html]

Details for gene set GOBP\_REGULATION\_OF\_DNA\_TEMPLATED\_TRANSCRIPTION\_IN\_RESPONSE\_TO\_STRESS[GSEA]

|  || Dataset | 55457\_collapsed\_to\_symbols.cls.cls #OA\_versus\_normal.cls.cls #OA\_versus\_normal\_repos |
| Phenotype | cls.cls#OA\_versus\_normal\_repos |
| Upregulated in class | normal |
| GeneSet | GOBP\_REGULATION\_OF\_DNA\_TEMPLATED\_TRANSCRIPTION\_IN\_RESPONSE\_TO\_STRESS |
| Enrichment Score (ES) | -0.48652202 |
| Normalized Enrichment Score (NES) | -1.8477905 |
| Nominal p-value | 0.0 |
| FDR q-value | 0.06964168 |
| FWER p-Value | 0.907 |
Table: GSEA Results Summary

  

Fig 1: Enrichment plot: GOBP\_REGULATION\_OF\_DNA\_TEMPLATED\_TRANSCRIPTION\_IN\_RESPONSE\_TO\_STRESS      
 Profile of the Running ES Score & Positions of GeneSet Members on the Rank Ordered List

  

| SYMBOL | TITLE | RANK IN GENE LIST | RANK METRIC SCORE | RUNNING ES | CORE ENRICHMENT || 1 | NCK1 | NCK adaptor protein 1 [Source:HGNC Symbol;Acc:HGNC:7664] | 222 | 0.804 | 0.0327 | No |
| 2 | NFE2L2 | "nuclear factor, erythroid 2 like 2 [Source:HGNC Symbol;Acc:HGNC:7782]" | 2034 | 0.357 | -0.0859 | No |
| 3 | CHEK1 | checkpoint kinase 1 [Source:HGNC Symbol;Acc:HGNC:1925] | 3173 | 0.233 | -0.1599 | No |
| 4 | RPS6KA1 | ribosomal protein S6 kinase A1 [Source:HGNC Symbol;Acc:HGNC:10430] | 3389 | 0.213 | -0.1634 | No |
| 5 | RELA | "RELA proto-oncogene, NF-kB subunit [Source:HGNC Symbol;Acc:HGNC:9955]" | 3942 | 0.161 | -0.1964 | No |
| 6 | TMBIM6 | transmembrane BAX inhibitor motif containing 6 [Source:HGNC Symbol;Acc:HGNC:11723] | 4045 | 0.152 | -0.1949 | No |
| 7 | VHL | von Hippel-Lindau tumor suppressor [Source:HGNC Symbol;Acc:HGNC:12687] | 4142 | 0.143 | -0.1935 | No |
| 8 | MAPK7 | mitogen-activated protein kinase 7 [Source:HGNC Symbol;Acc:HGNC:6880] | 4396 | 0.121 | -0.2056 | No |
| 9 | IMPACT | impact RWD domain protein [Source:HGNC Symbol;Acc:HGNC:20387] | 4635 | 0.101 | -0.2178 | No |
| 10 | TP53 | tumor protein p53 [Source:HGNC Symbol;Acc:HGNC:11998] | 5511 | 0.032 | -0.2839 | No |
| 11 | ANKRD2 | ankyrin repeat domain 2 [Source:HGNC Symbol;Acc:HGNC:495] | 6186 | -0.019 | -0.3352 | No |
| 12 | SIRT2 | sirtuin 2 [Source:HGNC Symbol;Acc:HGNC:10886] | 6279 | -0.026 | -0.3407 | No |
| 13 | BACH1 | BTB domain and CNC homolog 1 [Source:HGNC Symbol;Acc:HGNC:935] | 6909 | -0.073 | -0.3851 | No |
| 14 | RPS6KA3 | ribosomal protein S6 kinase A3 [Source:HGNC Symbol;Acc:HGNC:10432] | 7092 | -0.087 | -0.3939 | No |
| 15 | CITED2 | Cbp/p300 interacting transactivator with Glu/Asp rich carboxy-terminal domain 2 [Source:HGNC Symbol;Acc:HGNC:1987] | 7100 | -0.087 | -0.3890 | No |
| 16 | CREB3L1 | cAMP responsive element binding protein 3 like 1 [Source:HGNC Symbol;Acc:HGNC:18856] | 7324 | -0.104 | -0.3998 | No |
| 17 | NEDD4 | NEDD4 E3 ubiquitin protein ligase [Source:HGNC Symbol;Acc:HGNC:7727] | 7407 | -0.111 | -0.3993 | No |
| 18 | RGS14 | regulator of G protein signaling 14 [Source:HGNC Symbol;Acc:HGNC:9996] | 7719 | -0.135 | -0.4151 | No |
| 19 | HIF1AN | hypoxia inducible factor 1 subunit alpha inhibitor [Source:HGNC Symbol;Acc:HGNC:17113] | 7815 | -0.142 | -0.4136 | No |
| 20 | MUC1 | "mucin 1, cell surface associated [Source:HGNC Symbol;Acc:HGNC:7508]" | 7871 | -0.146 | -0.4088 | No |
| 21 | HSPA1A | heat shock protein family A (Hsp70) member 1A [Source:HGNC Symbol;Acc:HGNC:5232] | 7876 | -0.146 | -0.4000 | No |
| 22 | BCLAF1 | BCL2 associated transcription factor 1 [Source:HGNC Symbol;Acc:HGNC:16863] | 8193 | -0.170 | -0.4140 | No |
| 23 | ATF6 | activating transcription factor 6 [Source:HGNC Symbol;Acc:HGNC:791] | 8680 | -0.208 | -0.4389 | No |
| 24 | RBPJ | recombination signal binding protein for immunoglobulin kappa J region [Source:HGNC Symbol;Acc:HGNC:5724] | 8800 | -0.217 | -0.4347 | No |
| 25 | GCN1 | GCN1 activator of EIF2AK4 [Source:HGNC Symbol;Acc:HGNC:4199] | 9327 | -0.258 | -0.4596 | No |
| 26 | DNAJB1 | DnaJ heat shock protein family (Hsp40) member B1 [Source:HGNC Symbol;Acc:HGNC:5270] | 9380 | -0.262 | -0.4473 | No |
| 27 | ARNT | aryl hydrocarbon receptor nuclear translocator [Source:HGNC Symbol;Acc:HGNC:700] | 9427 | -0.267 | -0.4343 | No |
| 28 | DDIT3 | DNA damage inducible transcript 3 [Source:HGNC Symbol;Acc:HGNC:2726] | 9551 | -0.278 | -0.4266 | No |
| 29 | NCK2 | NCK adaptor protein 2 [Source:HGNC Symbol;Acc:HGNC:7665] | 9755 | -0.297 | -0.4239 | No |
| 30 | TCIM | transcriptional and immune response regulator [Source:HGNC Symbol;Acc:HGNC:1357] | 10534 | -0.377 | -0.4610 | Yes |
| 31 | HSPA5 | heat shock protein family A (Hsp70) member 5 [Source:HGNC Symbol;Acc:HGNC:5238] | 10820 | -0.407 | -0.4579 | Yes |
| 32 | HSF1 | heat shock transcription factor 1 [Source:HGNC Symbol;Acc:HGNC:5224] | 11189 | -0.456 | -0.4582 | Yes |
| 33 | MBTPS2 | "membrane bound transcription factor peptidase, site 2 [Source:HGNC Symbol;Acc:HGNC:15455]" | 11280 | -0.468 | -0.4361 | Yes |
| 34 | KLF2 | Kruppel like factor 2 [Source:HGNC Symbol;Acc:HGNC:6347] | 11350 | -0.478 | -0.4117 | Yes |
| 35 | BAG3 | BAG cochaperone 3 [Source:HGNC Symbol;Acc:HGNC:939] | 11562 | -0.509 | -0.3964 | Yes |
| 36 | EPAS1 | endothelial PAS domain protein 1 [Source:HGNC Symbol;Acc:HGNC:3374] | 11758 | -0.542 | -0.3779 | Yes |
| 37 | HIF1A | hypoxia inducible factor 1 subunit alpha [Source:HGNC Symbol;Acc:HGNC:4910] | 11833 | -0.556 | -0.3491 | Yes |
| 38 | ATF4 | activating transcription factor 4 [Source:HGNC Symbol;Acc:HGNC:786] | 11999 | -0.593 | -0.3251 | Yes |
| 39 | EGR1 | early growth response 1 [Source:HGNC Symbol;Acc:HGNC:3238] | 12052 | -0.607 | -0.2914 | Yes |
| 40 | ATF6B | activating transcription factor 6 beta [Source:HGNC Symbol;Acc:HGNC:2349] | 12171 | -0.636 | -0.2610 | Yes |
| 41 | HMOX1 | heme oxygenase 1 [Source:HGNC Symbol;Acc:HGNC:5013] | 12421 | -0.714 | -0.2360 | Yes |
| 42 | NOTCH1 | notch receptor 1 [Source:HGNC Symbol;Acc:HGNC:7881] | 12555 | -0.776 | -0.1981 | Yes |
| 43 | VEGFA | vascular endothelial growth factor A [Source:HGNC Symbol;Acc:HGNC:12680] | 12629 | -0.813 | -0.1533 | Yes |
| 44 | ATF3 | activating transcription factor 3 [Source:HGNC Symbol;Acc:HGNC:785] | 12659 | -0.833 | -0.1038 | Yes |
| 45 | CEBPB | CCAAT enhancer binding protein beta [Source:HGNC Symbol;Acc:HGNC:1834] | 12804 | -0.973 | -0.0544 | Yes |
| 46 | JUN | "Jun proto-oncogene, AP-1 transcription factor subunit [Source:HGNC Symbol;Acc:HGNC:6204]" | 12813 | -0.994 | 0.0068 | Yes |
Table: GSEA details [plain text format]

  

Fig 2: GOBP\_REGULATION\_OF\_DNA\_TEMPLATED\_TRANSCRIPTION\_IN\_RESPONSE\_TO\_STRESS      
 Blue-Pink O' Gram in the Space of the Analyzed GeneSet

  

Fig 3: GOBP\_REGULATION\_OF\_DNA\_TEMPLATED\_TRANSCRIPTION\_IN\_RESPONSE\_TO\_STRESS: Random ES distribution      
 Gene set null distribution of ES for **GOBP\_REGULATION\_OF\_DNA\_TEMPLATED\_TRANSCRIPTION\_IN\_RESPONSE\_TO\_STRESS**
